# Cardiovascular Risk Reduction with Renin-Angiotensin Aldosterone System Blockade

**DOI:** 10.1155/2010/101749

**Published:** 2010-08-12

**Authors:** Nancy Houston Miller

**Affiliations:** Stanford Cardiac Rehabilitation Program, 703 Welch Road, Suite F1, Palo Alto, CA 94304, USA

## Abstract

This paper examines the evidence supporting treatments within the renin-angiotensin aldosterone system (RAS), the role cardioprotection plays within the management of hypertension, considerations around medication adherence, and the role of the nurse or nurse practitioner in guiding patients to achieve higher hypertension control rates. A large body of data now exists to support the use of angiotensin receptor blockers (ARBs) and angiotensin-converting enzyme inhibitors (ACEIs) which act on RAS, in the management of hypertension and their effect on cardiovascular risk reduction. Current evidence suggests that inhibition of the RAS is an important target for cardioprotection. RAS inhibition controls blood pressure and also reduces target-organ damage. This is especially important in populations at high-risk for damage including patients with diabetes and those with chronic kidney disease. Both ARBs and ACEIs target the RAS offering important reductions in both BP and target organ damage.

## 1. Introduction

Nurse practitioners and nurses play a key role in the prevention and management of chronic conditions such as cardiovascular disease (CVD), diabetes mellitus, and kidney disease. Despite strides made in its treatment and prevention, CVD remains the leading cause of death worldwide [1]. Myocardial infarction (MI), stroke, and renal failure are its most common complications. In 2005, CVD was the underlying cause of 17.5 million deaths, or 30% of all deaths globally—nearly equal to the entire population of the state of Florida. MI accounted for 7.6 millions of those deaths and strokes for 5.7 millions [1]. In the United States, 631,636 died from heart disease, the number one cause of death, whereas 137,119 deaths occurred as the result of stroke and 45,344 as the result of kidney disease [2]. Stroke and kidney diseases are the third and ninth leading causes of death, respectively. 

The morbidity associated with CVD is high as well. Currently, approximately 24.1 million Americans have been diagnosed with heart disease, and this condition resulted in 2.4 million hospital discharges in 2005. Approximately 5.6 million Americans have at one time or another had a stroke, and in 2005, stroke accounted for 1 million hospital discharges. About 3.3 million Americans have been diagnosed with kidney disease [2]. The costs in terms of death, disability, reduced productivity or loss of income, and healthcare expense are enormous. US healthcare costs for CVD total more than $149 billions annually, or 17% of all medical expenditures [3].

Risks associated with CVD include increasing age, male gender, heredity, hypertension, smoking, high blood cholesterol, lack of physical activity, diabetes, and obesity [4]. Clearly, age, gender, and heredity cannot be altered. Other risk factors are modifiable, and actions such as smoking cessation, eating a healthier diet, and getting adequate exercise can reduce an individual's risk of developing CVD. Hypertension is the leading preventable risk factor. It has shown a continuous, consistent, and independent association with the risk of developing CVD [5]. However, control of hypertension remains less than optimal. Currently, only 1 in 3 patients with hypertension has achieved optimal blood pressure (BP) control [5].

## 2. Background

**Figure 1:**
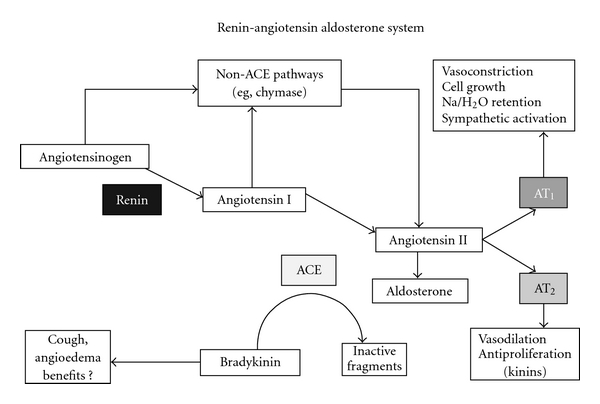
Renin-angiotensin aldosterone system. Reprinted with permission from Ibrahim [8].

The renin-angiotensin aldosterone system (RAS; Figure 1) is essential to the regulation of salt and water in the body [6, 7]. It is the RAS that maintains BP and vascular tone, primarily through signals from the kidney that are generated in response to changes in salt and water intake [6–8]. Although most of the RAS is based in the kidneys, there is tissue RAS as well [6, 7, 9]. The kidney or endocrine RAS is responsible for short-term volume and pressure adjustments, whereas the tissue RAS appears to affect long-term changes in the circulatory system [9, 10].

## 3. The RAS Cycle

The RAS cycle begins when angiotensinogen is produced in the liver and excreted. It is converted to angiotensin I by the enzyme renin, which is produced in the juxtaglomerular cells of the kidney. Angiotensin-converting enzyme (ACE) then converts angiotensin I to angiotensin II. Circulating angiotensin II activates AT_1_ receptors in a variety of target tissues, which results in increased water and sodium reabsorption, cell proliferation, and changes in vascular tone [7]. The consequences of these effects are an increase in blood volume and systemic vasoconstriction and a subsequent rise in BP [7, 8]. It is important to note that angiotensin II can be generated directly from angiotensinogen through non-ACE pathways, including cathepsin G, chymase, and ACE-2-dependent pathways [6, 8, 10]. These alternative pathways are responsible for persistent production of angiotensin II during ACE inhibition.

Angiotensin II binds to both AT_1_ and AT_2_ receptors. AT_1_ upregulates the sympathetic nervous system, increasing vasoconstriction, aldosterone release, and sodium retention [6, 8, 10, 11]. Angiotensin II also promotes the production of free radicals, stimulates plasminogen activator inhibitor-1 release, and increases tissue factor and vascular cell adhesion molecule expression [6]. Additionally, angiotensin II has proatherogenic effects through promotion of vascular smooth muscle cell proliferation and leukocyte adhesion, thus playing an important role in the development of CVD [6, 8]. Angiotensin II also reduces the beneficial vasodilatory effects of nitric oxide through inhibition of nitric oxide synthase [10]. However, in binding to the AT_2_ receptor, angiotensin II mediates apparent beneficial effects that counterbalance AT_1_ receptor stimulation [10].

## 4. The RAS in Hypertension and CVD

**Figure 2:**
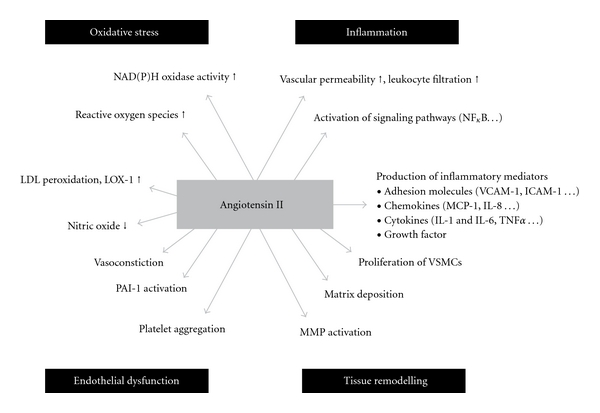
Important effects of angiotensin II on mechanisms associated with atherosclerosis. Reprinted with permission from Schmieder et al. [15]

Chronic elevation of RAS with subsequent exposure of tissues to high levels of angiotensin II results in hypertension, CVD, and target-organ damage. Hypertension creates stress on the blood vessel walls, giving rise to endothelial injury and thrombotic and inflammatory complications [12]. The vascular endothelium regulates blood fluidity and coagulation, vascular growth, inflammation, and vascular tone. These processes are primarily under the control of the renin-angiotensin and kallikrein-kinin systems [12, 13]. Bradykinin, a potent vasodilator, is degraded by ACE. In combination with the conversion of angiotensin I to angiotensin II, the reduction in bradykinin levels by ACE leads to enhanced vasoconstriction and inhibition of fibrinolysis [12, 14, 15] (Figure 2).

The risks of CVD presented by the disruption of vascular homeostasis in the face of hypertension are increased in patients with diabetes mellitus. More than 65% of individuals with diabetes die from heart disease or stroke, and their risk of death from heart disease is 2 to 4 times higher than that of nondiabetic adults, whereas the risk of death from stroke is 2.8 times higher [16]. Approximately 73% of adults with diabetes have hypertension, and diabetes accounts for 44% of new cases of kidney disease each year [16]. It is the most common reason for kidney transplantation [17].

## 5. The Role of Angiotensin Receptor Blockers and ACE Inhibitors: RAS Inhibition

 ACEIs and ARBs block the activity of the RAS in different ways. Whereas ACEIs prevent the formation of angiotensin II by inhibiting ACE, ARBs block the angiotensin II type 1 receptor, thus preventing angiotensin II formed by ACE and non-ACE pathways from binding to the AT_1_ receptor. ARBs also stimulate AT_2_ receptors [15, 18]. Interestingly, the AT_2_ receptor antagonizes many of the effects of the AT_1_ receptor, such as cell proliferation, and stimulation of the AT_2_ receptor appears to provide protection for certain organs, such as the brain against ischemia [15]. 

Long-term use of ACEIs can lead to secondary increases in angiotensin II and aldosterone through the secondary (non-ACE) pathways, also known as “ACE escape” [18]. Of the non-ACE pathways, the most important for the formation of angiotensin II is the chymase pathway [19]. Of significant interest, recent data suggest that the chymase pathway is upregulated in diabetic and hypertensive nephropathy, and thus ACE escape may be more marked in patients with renal disease [18, 19]. Chymase also has been found to be upregulated in the coronary vascular and kidney tissue of patients with diabetes in general [15, 20]. 

Although the phenomenon of ACE escape represents a drawback for the ACEI drug class in the treatment of hypertension, the ARB class is not without its own shortcomings. Treatment with ARBs may result in rebound concentrations of renin and angiotensin II by disrupting the negative feedback loop within the RAS [18]. The renal RAS has been shown to be separate from the systemic RAS, and doses of ARBs necessary to achieve adequate renal tissue concentrations to inhibit intrarenal RAS and prevent rebound of angiotensin II exceed those necessary to attain maximal BP-lowering effects [18]. Thus, it has been suggested that combination therapy with ACEIs and ARBs may provide the best option for patients with kidney disease, because some of these patients continue to progress to end-stage renal disease despite treatment with one or the other class as monotherapy [18].

The kidneys are not the only target organs at risk in patients with hypertension. Hypertension and upregulation of the RAS affect the heart, brain, and vascular endothelium as well, and there is evidence that blockade of the RAS can reduce damage to these target organs [15]. RAS activation has been noted to contribute to left ventricular hypertrophy in patients with primary hypertension independently of and in addition to the BP load exerted on the left ventricle [15]. The RAS may also play a role in the development of atrial fibrillation. RAS blockade by ARBs in animals has been shown to slow conductivity and to prevent left atrial dilation and fibrosis, suggesting that RAS blockade may be effective as a preventive and therapeutic strategy for atrial fibrillation [15]. Stroke is another important CVD complication, and hypertension contributes substantially to its risk. Good BP control is the most effective method of reducing this risk. However, meta-analyses indicate that ARBs provide benefit in stroke risk reduction that go beyond BP control [15]. Cerebral AT_2_ receptors exert neuroprotective effects in response to ischemic neuronal damage. Therefore, stimulation of these receptors by ARBs may prove more effective in stroke management than therapy with ACEIs [15, 21]. Atherosclerosis contributes to risk of coronary and cerebrovascular events. The binding of angiotensin II to the AT_1_ receptors appears to be central to the atherosclerotic cascade, implicating the RAS in endothelial dysfunction and the development of atherosclerosis. Evidence suggests that both ACEIs and ARBs improve endothelial function [15]. Finally, RAS blockade may reduce insulin resistance, which is characteristic of both the metabolic syndrome and type 2 diabetes mellitus. Data indicate that both ACEIs and ARBs may reduce the frequency of new-onset type 2 diabetes in hypertensive patients, in contrast to *β*-blockers and diuretics, which do not [15].

## 6. The Present Paper

A large body of data now exists to support the use of angiotensin receptor blockers (ARBs) and ACE inhibitors (ACEIs) in the management of hypertension [15, 17, 22, 23]. 

## 7. Trials of ACEIs

The Heart Outcomes Prevention Evaluation (HOPE) trial investigated the effects of the ACEI ramipril on cardiovascular (CV) events in 9,297 patients who had diabetes or evidence of CVD (coronary, cerebrovascular, or peripheral artery disease) and were therefore considered at high risk, but who did not have left ventricular dysfunction or heart failure (HF) [23]. Patients were randomly assigned to receive either ramipril 10 mg once daily or matching placebo for 5 years. The primary endpoint was a composite of MI, stroke, or CV-related death. 

The primary endpoint was reached by 14.1% (*n* = 651) of those receiving ramipril and 17.8% (*n* = 826) of those in the placebo group (*P* < .001). The relative risk was 0.78, and the upper bound of the 95% confidence interval (CI) of 0.70 to 0.86 includes at least a relative risk reduction of 14% [23]. Statistically significant reductions were also found for death from CV causes (6.1% for ramipril, 8.1% for placebo; relative risk, 0.74; *P* < .001), MI (9.9% versus 12.3%, resp.; relative risk, 0.80; *P* < .001), stroke (3.4% versus 4.9%, resp.; relative risk, 0.68; *P* < .001), and death from any cause (10.4% versus 12.2%; relative risk, 0.84; *P* < .005) [23]. Complications related to diabetes were significantly reduced as well (6.4% versus 7.6%, resp.; relative risk, 0.84; *P* < .03).

The findings of HOPE provided evidence-based support that ramipril is beneficial in a broad range of patients considered to be at high risk for CV events. Ramipril lowered the combined primary endpoint in the total patient population by 22%. The magnitude of benefit with ramipril was at least as great as that achieved with agents as *β*-blockers, aspirin, and lipid-lowering agents for secondary prevention over 4 years of treatment [23].

In the subgroup of patients with diabetes (38.5%; *n* = 3,577), the risk of the combined primary endpoint was significantly reduced by 25% (95% CI, 12–36; *P* = .0004), and progression to overt nephropathy was reduced by 24% (95% CI, 3–40; *P* = .027) [23, 24]. 

EUROPA (European trial On reduction of cardiac events with Perindopril in patients with stable Artery disease) examined the use of another ACEI, perindopril, in 13,655 patients with stable coronary artery disease, including 64% with a previous MI, 61% with angiographic evidence of coronary artery disease, 55% with coronary revascularization, and 5% whose only evidence of coronary artery disease was a positive stress test. After a preliminary run-in period of 5 weeks, during which all patients received perindopril, patients were randomized to perindopril 8 mg once daily (*n* = 6,110) or matching placebo (*n* = 6,108). The primary outcome measure was time to first occurrence of CV death, MI, or cardiac arrest [25]. Patients also received other agents known to reduce CV risk, including *β*-blockers, aspirin, and lipid-lowering agents [25].

The mean follow-up was 4.2 years. The primary endpoint was experienced by 8% of those receiving perindopril and 10% of those on placebo, for a 20% relative risk reduction in favor of perindopril (95% CI, 9–29; *P* = .0003). The investigators concluded that in patients with stable coronary heart disease and without apparent HF, 50 patients would need to be treated with perindopril for 4 years to prevent one major CV event [25].

The Prevention of Events with Angiotensin Converting Enzyme Inhibition (PEACE) trial investigated an ACEI, trandolapril, in 8,290 patients with stable coronary artery disease. Patients were randomized to either trandolapril 4 mg per day or matching placebo; 72% of patients had previously undergone coronary revascularization and 70% received lipid-lowering drugs during the trial period [26]. The primary endpoint was death from CV causes, MI, or coronary revascularization. Over 4.8 years, this outcome occurred in 21.9% of those receiving trandolapril and 22.5 of those receiving placebo (hazard ratio, 0.96; 95% CI, 0.88–1.06; *P* = .43). This study indicated that the addition of an ACEI provides no further benefit in terms of death from CV causes, MI, or coronary revascularization [26].

## 8. Trials of ARBs

ARBs have also figured prominently in recent clinical trials. The Candesartan in Heart Failure: Assessment of Reduction in Mortality and Morbidity (CHARM)-Alternative study looked at candesartan therapy in patients with chronic HF and reduced left-ventricular systolic function who were intolerant to ACEIs. A total of 2,028 patients with symptomatic HF and a left-ventricular ejection fraction of 40% or less were randomized to receive a targeted dose of candesartan 32 mg once daily or matching placebo. The primary endpoint was the composite of CV death or hospital admission for chronic HF [27]. 

Over a mean follow-up of 33.7 months, 33% of the patients receiving candesartan and 40% of those receiving placebo experienced the primary endpoint (hazard ratio, 0.77; 95% CI, 0.67–0.89; *P* = .0004), resulting in a 23% relative risk reduction with candesartan. Importantly, permanent discontinuation of study drug was similar in the candesartan (30%) and placebo (29%) groups [27].

Valsartan also was investigated in patients with chronic HF. A total of 5,010 chronic HF patients already receiving pharmacologic therapy considered optimal by their physicians (93% were on an ACEI at baseline) were randomly assigned to valsartan 160 mg twice daily or matching placebo. The primary endpoints were mortality and the combined endpoint of mortality and morbidity, defined as cardiac arrest with resuscitation, hospitalization for HF, or receipt of inotropic or vasodilator therapy for 4 hours or more [28].

Although overall mortality was similar in both groups, the combined endpoint was 13.2% lower in the valsartan group than with placebo (relative risk, 0.87; 97.5% CI, 0.77–0.97; *P* = .009). This latter result was primarily driven by a lower incidence of patients hospitalized for HF in the valsartan group compared with placebo (13.8% versus 182%, resp.; *P* < .001). Treatment with valsartan was also associated with improvement in New York Heart Association class, ejection fraction, signs and symptoms of HF, and quality of life as compared with placebo (*P* < .01). Thus, valsartan proved to be valuable when added to prescribed therapy in patients with HF. However, a post hoc analysis of a subgroup of patients receiving a combination of valsartan, an ACEI, and a *β*-blocker had an increase in mortality and morbidity, suggesting that not all combinations improve patient outcomes [28].

The Ongoing Telmisartan Alone and in Combination with Ramipril Global Endpoint Trial (ONTARGET) was conducted in patients with vascular disease or high-risk diabetes without HF to determine if the ARB, telmisartan, would be as effective as the ACEI, ramipril; and whether a combination of both agents would be superior to ramipril alone. Patients were randomized to ramipril 10 mg daily (*n* = 8,576), telmisartan 80 mg daily (*n* = 8,542), or a combination of both agents (*n* = 8,502). The primary composite endpoint was death from CV causes, MI, stroke, or hospitalization for HF [27].

At a median follow-up of 56 months, the primary endpoint was reached by 16.5% of those in the ramipril group and 16.7% in the telmisartan group (relative risk, 1.01; 95% CI, 0.94–1.09). The telmisartan group had lower incidence of cough and angioedema and a higher incidence of hypotensive symptoms associated with permanent discontinuation of study medication compared with the ramipril group. The investigators concluded that telmisartan was as effective as ramipril in reducing the risk for CV death/MI/stroke and hospitalization for HF in this high-risk patient population [27].

## 9. Trials of Combination Therapy with ACEIs and ARBs

Because ACEIs and ARBs inhibit the RAS in different and potentially complementary ways, it was thought that combination therapy with these 2 drug classes might prove beneficial in preventing or mitigating target-organ damage in patients with hypertension. The CHARM-Added study evaluated the efficacy of candesartan in patients with chronic HF and reduced left-ventricular systolic function. A total of 2,548 patients were randomized to either a targeted dose of 32 mg of candesartan once daily or placebo in addition to concurrent ACEI therapy. The primary outcome was the composite of CV death or admission to hospital for chronic HF [29]. 

Over a median follow-up of 41 months, 38% of patients receiving candesartan and 42% receiving placebo experienced a primary outcome event. The hazard ratio was 0.85 (95% CI, 0.75–0.96; *P* = .011), significantly favoring candesartan versus placebo. The annual event rates were 14.1% in the candesartan group and 16.6% in the placebo group [29]. CHARM-Added showed that in patients with chronic HF and a low left-ventricular ejection fraction, the addition of candesartan to an ACEI led to further reductions in the risk of CV-related mortality and hospital admission for chronic HF [29].

In the Valsartan in Acute Myocardial Infarction Trial (VALIANT), the efficacy of monotherapy with valsartan, captopril, or the combination of the 2 was explored in patients who had experienced an acute MI. Within 0.5 to 10 days after the event, patients were randomized to valsartan (4,909 patients), captopril (4,909 patients), or the combination (4,885 patients). The primary study outcome was death from any cause [30].

At a median follow-up of 24.7 months, 19.9% of patients receiving valsartan, 19.5% of patients receiving captopril, and 19.3% of patients receiving combination therapy had died. These differences were not significant, and valsartan was found to be noninferior to captopril (*P* = .004), but no benefit was found for the combination therapy for this endpoint. However, drug-related adverse effects were more common with the combination of valsartan and captopril than in either monotherapy group [30].

ONTARGET found that the combination of telmisartan plus ramipril was not superior to ramipril alone. The primary outcome of CV death, MI, stroke, or hospitalization for HF occurred in 16.3% of patients receiving combination therapy, as compared with 16.5% in those receiving ramipril (relative risk, 0.99; 95% CI, 0.92–1.07). The combination resulted in significantly higher incidence of hypotensive symptoms, syncope, and renal dysfunction compared with ramipril alone [27].

The results of ONTARGET suggest that combining 2 distinct classes of agents that inhibit the RAS at different sites does not improve patient outcomes in a broad spectrum of high-risk subjects without HF. It corroborates the findings of VALIANT and is in contrast to the findings of CHARM-Added. However, it must also be noted that the ONTARGET patient population and that of VALIANT differ fundamentally, because the latter trial was conducted in patients who had experienced an acute MI and had signs of HF, radiographic evidence of left ventricular systolic dysfunction, or both.

## 10. Adherence with RAS Agents

While the use of RAS agents has shown some significant benefit within controlled studies, there continues to be a struggle with many patients taking their prescribed medication to achieve maximum outcomes. There are no recent studies specific to nurses and their role within the management of RAS agents relative to a patient's adherence to medications; however, several factors can be seen through other studies. A recent study within hypertension has shown that medication adherence was significantly associated with systolic blood pressure (*r* = .253, *P* < .04), thus, prompting the need for strict adherence to prescribed regimens [31]. This adherence can be increased with a long-term intervention from health professionals as seen in the VALIDATE Study; however, when such an intervention stops, adherence declines as well [32]. This decline points out that a healthcare provider's intervention is only one component in increasing adherence. In a 2005 HealthStyles survey of 1432 individuals who received prescriptions for antihypertensive medications, 407 (28.4%) reported having difficulty taking their medication. “Not remembering” was the most common reason reported (32.4%), but cost (22.6%), having no insurance (22.4%), side effects (12.5%), and not thinking there is any need (9.3%) were also important indicators. Additionally younger age, lower income, having mental function impairment, and having had a blood pressure check more than 6 months earlier were factors significantly associated with nonadherence. While utilizing the right medications to decrease the risk of cardiovascular disease is vital, alleviating barriers to medication adherence should be a major goal within management [33].

## 11. Discussion

ARBs are a proven option in patients with hypertension, particularly those who are at risk for target-organ damage, such as those with diabetes or evidence of CVD. Evidence from clinical trials demonstrates that these agents provide good control of hypertension and reduce CV risk. To this end, some ACEIs and ARBs have received FDA-approved indications to reduce CV risk. Adverse effects associated with use of ACEIs include cough, rash, taste disturbance, and angioedema. Cough, angioedema, taste disturbance, and rash occur less frequently with ARBs than with ACEIs. However, hypotension is more common with ARBs than ACEIs [30]. 

The side-effect profile of ARBs may lead to better adherence on the part of patients. Adherence is notoriously poor with hypertension. Nonadherence to hypertension therapy is influenced by misunderstanding of the condition or treatment, denial of illness because of lack of symptoms, lack of patient involvement in the care plan, or unexpected adverse effects of medications. Many of these contribute to the 34% of hypertension patients who are not adequately controlled with their current treatment regimen [5]. 

However, adherence is an area in which nurses and nurse practitioners can have a positive impact. Patients must be motivated to take their medication as prescribed, and to do so, they must understand the importance of doing so. Patient motivation is enhanced through education, positive experiences with the healthcare system, and trusting in the nurses and nurse practitioners who oversee medical care. Empathy by all healthcare professionals is a powerful motivator [5]. Patients must agree on BP goals, and the cost of medications and the complexity of the regimen must be taken into account. Patients must also be clear about their responsibility to adhere to the regimen and must make sensible lifestyle changes [5].

## 12. Conclusions

Inhibition of the RAS is an important target for cardioprotection. RAS inhibition not only controls BP, but it also reduces target-organ damage. This is especially important in populations at high risk for such damage, such as diabetics and those with chronic kidney disease. Both ARBs and ACEIs effectively control the RAS, offering important reductions in both BP and target-organ damage.

## 13. Relevance to Clinical Practice

Cardiovascular protection is a key element in the overall management of hypertension. Since the risk of CVD doubles with each increment of 20/10 mmHg, there should be some care given to selecting the right agent for an individual [5]. Due to the overwhelming positive data on the inhibition of the RAS, selection of an agent in these classes should be considered. However, other factors such as patient co-morbidities, adherence, and risk for potential adverse drug events must also be considered when selecting an agent. Depending on the patient's needs, the use of ARBs or ACEIs can be used to effectively inhibit the RAS, offering important reductions in both BP and target-organ damage. Before selecting which agent to use to manage hypertension, consideration should be given to both classes' agents with respect to their FDA-approved indications.
